# Do mental or somatic diagnoses influence emotional response and perception of physician-assisted suicide in Germany? A vignette-based experiment

**DOI:** 10.1186/s12910-025-01223-3

**Published:** 2025-05-15

**Authors:** Laura Hofmann, Birgit Wagner

**Affiliations:** Department of Clinical Psychology, Medical School Berlin, Berlin, Germany

**Keywords:** Assisted suicide, Physician-assisted suicide, Suicide, Suicide prevention

## Abstract

**Background:**

Physician-assisted suicide (PAS) is increasingly being legalized in a growing number of countries and is the focus of societal and ethical debates. However, there is limited knowledge regarding the perception and acceptance of PAS across different physical and mental health conditions. This study aimed to explore emotional responses, understanding, and willingness to support individuals with the wish for PAS.

**Methods:**

Participants from the general German population (*N* = 512) were presented with four case vignettes of PAS depicting individuals in an online study: one with cancer, one with depression, one with schizophrenia, and one healthy individual. Participants were asked to evaluate the emotional reactions elicited by the desire for PAS, the extent of their understanding of this wish, and their willingness to support each individual.

**Results:**

The study revealed significant differences in reactions to the case vignettes. Pro-social emotions were lowest and anger highest when considering the healthy individual. Participants demonstrated the greatest understanding and highest willingness to support the individual with cancer, while the least understanding and support were observed for the healthy person.

**Conclusions:**

The differential levels of support for PAS across various conditions underscore the complex interplay between societal values, perceived quality of life, and ethical considerations, particularly when mental health is involved.

**Supplementary Information:**

The online version contains supplementary material available at 10.1186/s12910-025-01223-3.

## Introduction

Physician-assisted suicide (PAS) is a highly relevant and widely discussed topic, consistently highlighting the ethical dilemmas and challenges of managing end-of-life care. Proponents argue that PAS offers a long-awaited option for individuals to die in a self-determined manner, thus preserving their autonomy and dignity [1, 2]. Conversely, critics raise concerns about rapid accessibility, potential neglect of palliative care, and the coercion of vulnerable individuals [3, 4].

PAS has been permitted in Germany since the decision of the German Federal Constitutional Court in 2020 [5]. According to the Federal Constitutional Court (2020), the right to self-determined dying is not restricted to externally defined conditions such as severe or incurable illnesses. Individuals seeking PAS must be capable of providing informed consent and articulating their reasons in a clear and understandable way. PAS is provided by doctors and right-to-die organizations. Still, there are ongoing debates about a need for new regulations. Precise statistics on deaths resulting from PAS in Germany are unavailable due to the lack of comprehensive documentation. PAS involves a physician providing lethal medication, which the individual must self-administer [6]. Euthanasia remains illegal in Germany.

However, PAS is associated with numerous concerns, criticisms and fears. There seems to be a consensus among both the public and healthcare professionals regarding the groups for whom PAS should be accessible. PAS is more commonly accepted and deemed appropriate for individuals with terminal illnesses characterized by significant suffering and impending death, as well as for those with progressive diseases [7, 8].

Several studies have already examined attitudes on PAS in doctors, nurses or the general population. Studies from Norway showed that only about one-third of doctors support PAS for terminal illnesses, with just 12.7% endorsing it for chronic illnesses [9]. In contrast, the Norwegian general population shows greater acceptance of PAS, although they remain critical for chronic illnesses and even more for mental health disorders [7]. Generally, doctors’ perspectives on PAS differ from those of the general public, as they could be directly involved in PAS and have a distinct understanding of clinical diagnoses and treatment options. In addition, studies found that age, socio-economic-status, education level and religiosity can influence one’s attitudes towards PAS [3, 10].

However, opinions are divided on the accessibility of PAS for individuals with mental health disorders, chronic conditions, or no illness at all, with some advocating for restricted access in these cases. A study from Switzerland assessed the attitudes of 457 psychiatrists towards PAS for mental health disorders using case vignettes [11]. Approximately half of the psychiatrists rejected PAS for severe mental health disorders. Case vignettes were featuring patients with anorexia nervosa, treatment-resistant depression, and schizophrenia. The results showed some variation, with approximately one-third of respondents supporting PAS for the patients in each scenario.

The implementation of PAS for mental health disorders is generally associated with numerous concerns. Mental health disorders are believed to impair the formation of free will, and the wish for PAS may not be stable and individuals may opt for PAS too hastily [11, 12, 13]. In a study from New Zealand, 46% of respondents from the general population indicated that the presence of a mental health disorder should be an exclusion criterion for PAS [8]. While PAS for mental health disorders is only permitted in some countries, the requirements for eligibility are similar. The prerequisites include free will, unrestricted judgment, and an independent and autonomous decision [14, 15]. However, it is not clear whether these criteria can be met in the presence of mental health disorders. It is essential to ensure that the wish to die is not primarily a symptom of the existing disorder.

Some studies extend beyond surveying attitudes towards PAS and investigate emotional reactions, stigma, and the desire for distance from individuals seeking PAS, depending on the type of death or illness involved. One such study explored variations in these reactions based on the type of death (PAS vs. long-term illness) and age (28 vs. 80 years) [16]. Interestingly, the study found no significant differences in emotional reactions or the desire for social distance between the two types of death.

However, to date, no study has examined the perception of PAS across different diseases in Germany. This study aimed to assess the attitudes of the general population towards PAS in individuals with physical or mental illnesses as well as in healthy individuals using case vignettes. Participants rate their emotional reactions and the degree of support and understanding towards the person who wishes to die. The aim was to find out whether understanding and willingness to provide support depended on the presence of an illness. Based on existing research and theoretical frameworks, we hypothesized that participants would show fewer negative emotions and a higher level of positive emotions and understanding towards a person with a terminal physical illness. Previous studies have shown that individuals with terminal physical conditions are often seen as suffering in ways that evoke understanding for PAS. People tend to view mental illness as more treatable, which may result in a rejection of PAS in these cases [17, 18].

We also hypothesized that the least understanding and more negative emotions will be shown towards a healthy person with a wish for PAS. This study seeks to contribute to the broader discourse on PAS by elucidating how specific illnesses influence public perception and emotional response. This hypothesis is based on the assumption that healthy individuals seeking PAS will evoke less understanding, as they do not suffer from an illness. Additionally, there is likely to be less willingness to support healthy individuals in their desire for PAS [18, 19].

## Methods

### Design and sample characteristics

The study employed a cross-sectional design with participants completing an online questionnaire. Inclusion criteria were: (1) 18 years or older, (2) sufficient knowledge of German, and (3) signed informed consent. A sample size calculation was conducted to ensure the study was adequately powered. The sample size was calculated using G*Power with the following parameters: effect size *f* = 0.15; α-error = 0.05, power: 0.80, number of groups = 1, number of measurements = 4, resulting in a total sample of at least 62 participants [20]. A total of 799 individuals responded to the questionnaire, with complete data available from 562 participants. A total of 44 individuals had to be excluded due to missing data and another 6 participants due to outliers (> 3 SDs above the mean), resulting in a total sample of *N* = 512 participants. The outliers were based on survey duration to remove potentially inattentive or non-serious responses. Recruitment took place via social media (Facebook, Instagram) using a post that provided information about the study. The research group’s existing social media channels were used for this purpose. While efforts were made to reach a diverse sample, participation was voluntary, which may have influenced the composition of the final sample. Data were collected between July 25th and September 06th 2023. Data collection was conducted over a period of six weeks. The process was terminated at the end of this period, regardless of whether the pre-determined sample size had been reached, in order to ensure a representative time frame for the data collection. The Ethics Committee of the Medical School Berlin approved the study on July 12, 2023 (MSB-2023/117). A publication displaying attitudes on PAS while considering a possible influence of one’s own experience of loss from this study has already been published [21].

### Measures

#### Vignettes

We developed four distinct vignettes, each featuring a fictional individual. These vignettes detailed the person’s background, life circumstances, and health status. All four individuals were depicted as planning to utilize PAS. The scenarios included a person with cancer, a person with depression, a person with a schizophrenia spectrum disorder, and a person without any illness. The vignettes were collaboratively developed by the two authors, reviewed for clarity and accuracy by a third party, and subsequently revised. The vignettes can be accessed in the Supplementary Material.

#### Questionnaires

##### Sociodemographic information

The following demographic data was collected: Age, gender, relationship status, living situation and level of education. Participants were also asked whether they had lost someone to suicide and whether they had any personal experience with PAS.

##### Emotional reactions

The emotional reactions pro-social emotions, fear and anger were assessed using a total of 13 items. The items were originally based on the study by von dem Knesebeck [22] and were revised by Eisma [23] due to their low reliability. Therefore, one item of the pro-social emotions scale was deleted and a total of five items were added to the subscales. This resulted in the scales pro-social emotions with four items (e.g. “I feel pity.“), fear with five items (“I feel tense.“) and anger with four items (“I feel annoyed.“). The items are rated on a 4-point Likert Scale from 1 = *completely disagree* to 4 = *completely agree*. A sum score can be calculated for each factor. The internal consistency of the subscales in Eisma’s study [23] was acceptable with Cronbach’s α = 0.85 for the fear subscale, α = 0.82 for the anger subscale, and α = 0.75 for the pro-social emotions subscale. In this sample, the internal consistency was acceptable to excellent with α = 0.91, α = 0.72, and α = 0.71, respectively.

### Understanding and willingness to support

Understanding and willingness to support PAS were each assessed using six items, which were designed specifically for this study to capture distinct aspects of the respective constructs. Due to this multidimensional structure, item-level analyses were conducted to examine potential differences across these facets. Internal consistency for each item set was low (α = 0.48 and α = 0.47) supporting the relevance of disaggregated interpretations. The English version of the items can be accessed in the Supplementary Material.

Six items were used to measure how understandable participants consider the wish for assisted suicide to be (e.g., “I do not find Mrs. Meier’s illness serious enough to make use of assisted suicide.”). The items are rated on a 5-point Likert Scale from 1 = *completely disagree* to 5 = *completely agree* and are only evaluated on a descriptive level; no sum score is calculated. Aggregating the items could mask the subtle differences between these two constructs and therefore was not pursued.

A further six items recorded the extent to which participants would support the person in the case vignettes in their wish for assisted suicide (e.g., “If Mrs. Meier were a relative or friend, I would support her in her wish.”). The items are as well rated on 5-point Likert Scale from 1 = *completely disagree* to 5 = *completely agree*, no sum score is calculated.

### Statistical analyses

Analyses were conducted using SPSS Version 28. For continuous variables, means and standard deviations were calculated, while frequencies and descriptive statistics were computed for categorical variables. Normal distribution for the dependent variables was tested using the Kolmogorv-Smirnov test. Outliers were identified using box plots and were defined as > 3 SDs above the mean. These outliers were removed from the data set. In order to analyze the differences in the emotional reactions to the respective case vignettes, repeated measures ANCOVAs were calculated. Differences in acceptance and willingness to provide support in the various case vignettes were analyzed using further repeated measures ANCOVAs. To control for the influence of a personal experience of loss through suicide, this variable was included as a covariate. The Bonferroni adjustment was performed to correct for alpha error accumulation. We opted for an item-level-analysis for willingness to support and understanding of PAS. The aim was to explore the distinct dimensions these items were designed to capture which would be overseen when using a sum score. Given the exploratory nature of the study and the conceptual heterogeneity of the items, we chose not to aggregate responses into composite scores. This approach allowed for a more nuanced examination of participant attitudes.

## Results

### Sample characteristics

Of the 512 participants, 93.0% were female. The age of the participants ranged from 19 to 75 years (*M* = 44.36; *SD* = 10.96). Participants generally had a higher education, the education level was divided into secondary (high school, secondary school), higher secondary (vocational school) and tertiary education (university or PhD). Table 1 shows all sample characteristics. Participants with suicide loss and participants without suicide loss only differed significantly in terms of age, with people without suicide loss being slightly younger (*M* = 43.66, *SD* = 10.74) than people with suicide loss (*M* = 45.79, *SD* = 11.29), *t*(283.55) = 0.91, *p* =.043. Only two people stated that they had personal experience with PAS and both had lost someone to assisted suicide. However, as these two people had to be excluded due to missing data, this information was not included in the analyses.

**Table 1:** Sociodemographic data (*N* = 512)

	M (SD)/ *n*	Range/ %
**Age**	44.36 (10.96)	19–75
**Gender (female)**	476	93.0
**Marital status**		
Single In a relationship Married Divorced Widowed	83 89 253 54 33	16.2 17.4 49.4 10.5 6.4
**Education level**		
Secondary Higher Secondary Tertiary Other	56 270 182 2	11.0 52.7 35.5 0.4
**Suicide Loss (yes)**	168	32.8

### Emotional reactions

First, repeated measures ANCOVAs were conducted to analyze the differences in emotional reactions towards the case vignettes. The Greenhouse–Geisser adjustment was used to correct for violations of sphericity. While controlling for loss through suicide, significant differences emerged for pro-social emotions, *F*(2.55,1299.70) = 87.68, *p* <.001, partial η² = 0.15, for fear, *F*(2.90,1476.52) = 8.03, *p* <.001, partial η² = 0.02, and anger, *F*(1.99,1015.65) = 118.25, *p* <.001, η² = 0.19. Participants showed the highest expression of pro-social emotions in the depression vignette and the lowest in the healthy vignette. The highest level of fear was found in the depression vignette and the lowest in the healthy vignette. Anger was highest in the healthy vignette and lowest in the cancer vignette.

In order to analyze the emotional reactions more precisely, the individual ratings of the case vignettes were compared with each other in a post-hoc analysis while controlling for suicide loss. Table 2 shows means and standard deviations for the emotional reactions. Bonferroni-adjusted post-hoc analysis revealed significantly lower levels of pro-social emotions (*M*_Diff_ = -0.32, 95%-CI[-0.57, -0.07], *p* =.005), fear (*M*_Diff_ = -0.88, 95%-CI[-1.29, -0.47], *p* <.001), and anger (*M*_Diff_ = -0.67, 95%-CI[-0.89, -0.45], *p* <.001) for the cancer vignette compared to the depression vignette. Participants showed significantly higher levels of pro-social emotions (*M*_Diff_ = 1.69, 95%-CI[1.35, 2.03], *p* <.001) and lower levels of anger (*M*_Diff_ = -2.25, 95%-CI[-2.61, -1.90], *p* <.001) towards the cancer vignette compared to the healthy vignette. We also found significantly lower expression of pro-social emotions (*M*_Diff_ = -0.28, 95%-CI[-0.55, -0.01], *p* =.036) and lower levels of anger (*M*_Diff_ = -0.31, 95%-CI[-0.49, -0.13], *p* <.001) in the cancer vignette compared to the schizophrenia vignette.

**Table 2:** Differences in emotional reactions between the case vignettes (*N* = 512)

	M (SD)		M_Diff_	95%-CI		*p*
				Lower	Upper	
**Cancer - Depression**						
Anger	4.45 (1.22)	5.11 (2.05)	-0.67	-0.89	-0.45	**< 0.001**
Fear	9.27 (4.37)	10.15 (5.00)	-0.88	-1.29	-0.47	**< 0.001**
Pro-social	13.03 (2.64)	13.35 (2.79)	-0.32	-0.57	-0.07	**0.005**
**Cancer– healthy**						
Anger	4.45 (1.22)	6.70 (3.27)	-2.25	-2.61	-1.90	**< 0.001**
Fear	9.27 (4.37)	9.21 (4.74)	0.06	-0.37	0.49	1.00
Pro-social	13.03 (2.64)	11.34 (3.34)	1.69	1.35	2.03	**< 0.001**
**Cancer– schizophrenia**						
Anger	4.45 (1.22)	4.76 (1.70)	-0.31	-0.49	-0.13	**< 0.001**
Fear	9.27 (4.37)	9.51 (4.88)	-0.24	-0.68	0.20	0.872
Pro-social	13.03 (2.64)	13.31 (2.86)	-0.28	-0.55	-0.01	**0.36**
**Depression– Healthy**						
Anger	5.11 (2.05)	6.70 (3.27)	-1.59	-1.93	-1.25	**< 0.001**
Fear	10.15 (5.00)	9.21 (4.74)	0.95	0.52	1.37	**< 0.001**
Pro-social	13.35 (2.79)	11.34 (3.34)	2.01	1.68	2.34	**< 0.001**
**Depression - Schizophrenia**						
Anger	5.11 (2.05)	4.76 (1.70)	0.36	0.16	0.55	**< 0.001**
Fear	10.15 (5.00)	9.51 (4.88)	0.64	0.26	1.02	**< 0.001**
Pro-social	13.35 (2.79)	13.31 (2.86)	0.04	-0.19	0.26	**< 0.001**
**Healthy - Schizophrenia**						
Anger	6.70 (3.27)	4.76 (1.70)	1.94	1.62	2.27	**< 0.001**
Fear	9.21 (4.74)	9.51 (4.88)	-0.31	-0.69	0.08	0.205
Pro-social	11.34 (3.34)	13.31 (2.86)	-1.97	-2.31	-1.64	**< 0.001**

Note. two-tailed significant, Bonferroni-adjusted

In addition, participant showed significantly less anger (*M*_Diff_ = -1.59, 95%-CI[-1.93, -1.25], *p* <.001), more fear (*M*_Diff_ = 0.95, 95%-CI[0.52, 1.37], *p* <.001), and a significantly higher expression of pro-social emotions (*M*_Diff_ = 2.01, 95%-CI[1.68, 2.34], *p* <.001) in the depression vignette compared to the healthy vignette. When comparing the depression and the schizophrenia vignettes, analyses revealed significantly higher levels of anger (*M*_Diff_ = 0.36, 95%-CI[0.16, 0.55], *p* <.001) and fear (*M*_Diff_ = 0.64, 95%-CI[0.26, 1.02], *p* <.001) towards the depression vignette.

Participants also showed a significantly higher expression of anger (*M*_Diff_ = 1.94, 95%-CI[1.62, 2.27], *p* <.001) and lower pro-social emotions (*M*_Diff_ = -1.97, 95%-CI[-2.31, -1.64], *p* <.001) towards the healthy vignette compared to the schizophrenia vignette.

### Understanding and willingness to support

To assess participants’ understanding of the wish for PAS and their level of support for the individual described in the case vignette, we conducted an analysis of item characteristics, along with mean comparisons and differences between case vignettes, using repeated measures ANCOVAs. The Greenhouse–Geisser adjustment was again used to correct for violations of sphericity. The results of these analyses are presented in Table 3. Significant differences were observed across all items in the evaluation of the vignettes. Participants exhibited the least understanding of the wish for PAS in the vignette featuring a healthy individual. Furthermore, participants did not perceive the symptoms of the healthy individual as sufficiently severe for PAS, and opposed its permission for this individual. Participants expressed the lowest level of agreement with allowing PAS in the case of the healthy individual vignette. In contrast, participants demonstrated the highest level of understanding across all six items for the individual with cancer. They rated the wish for PAS as most comprehensible, perceived the symptoms as sufficiently severe to justify PAS, supported its permissibility for this group, and indicated that they would consider PAS themselves in a similar situation.

**Table 3:** Differences in understanding and willingness to support between the case vignettes (*N* = 512)

	Cancer	Depression	Healthy	Schizophrenia	F	*p* ^1^	η²
I can understand his/her wish for assisted suicide.	4.68 (0.73)	4.19 (1.10)	2.85 (1.50)	4.51 (0.86)	289.39	0.012	0.36
I think he/she should make use of further therapy options.	2.53 (1.28)	4.03 (1.11)	4.42 (0.98)	3.69 (1.29)	301.28	0.012	0.37
I do not find his/her illness/complaints serious enough to make use of assisted suicide.	1.44 (0.90)	2.40 (1.28)	3.62 (1.43)	2.06 (1.22)	279.32	0.012	0.35
He/she should not be allowed to die through assisted suicide.	1.28 (0.75)	2.35 (1.35)	3.34 (1.47)	2.04 (1.22)	254.85	0.012	0.33
I think assisted suicide should generally be allowed for people with this diagnosis.	4.24 (1.16)	2.96 (1.42)	2.17 (1.32)	3.31 (1.40)	273.90	0.012	0.35
If I were in his/her situation, I would also think about assisted suicide.	4.25 (1.13)	3.64 (1.29)	2.09 (1.27)	4.09 (1.06)	398.11	0.012	0.44
I would support him/her in his/her plans.	4.21 (1.05)	3.00 (1.32)	2.13 (1.19)	3.52 (1.24)	389.91	0.012	0.43
I would propose alternatives.	2.89 (1.29)	4.04 (1.10)	4.55 (0.81)	3.77 (1.19)	248.02	0.012	0.33
I would be furious about his/her decision	1.79 (1.15)	2.32 (1.35)	2.91 (1.52)	1.81 (1.15)	110.57	0.012	0.18
I would advise him/her not to do this.	1.75 (1.01)	2.99 (1.39)	3.82 (1.30)	2.59 (1.34)	306.46	0.012	0.38
I would accompany him/her to counseling sessions.	4.74 (0.66)	4.74 (0.67)	4.55 (0.92)	4.70 (0.71)	10.55	0.012	0.02
I would distance myself from him/her because of his/her wish.	1.06 (0.30)	1.23 (0.64)	1.37 (0.78)	1.17 (0.54)	24.51	0.012	0.05

Note. two-tailed significant, ^1^Bonferroni-adjusted

A similar trend was observed in the items assessing the level of support. Participants showed a higher willingness to support the individual with cancer, followed by the individual with a schizophrenia spectrum disorder. They were least likely to advise the person with cancer against the decision or to suggest alternative options. In contrast, support for the healthy individual was lowest; participants were more likely to suggest alternatives, express anger about the decision, and advise against it. Interestingly, while there were statistically significant differences in participants’ willingness to accompany the person to counseling or to distance themselves, these differences were minimal. This suggests that, despite varying levels of understanding, there remains a general willingness to offer support.

## Discussion

The aim of this study was to assess emotional reactions, understanding as well as support towards different medical conditions. We hypothesized that a request for PAS from an individual with a terminal physical illness is perceived as more understandable, elicits greater willingness to provide support, and is associated with fewer negative emotions. We expected the opposite reactions towards a request from a healthy individual.

Firstly, we found significant differences between all emotions assessed towards the different vignettes. Pro-social emotions were lowest, and anger was highest towards the healthy vignette. Participants also showed lower levels of anger, fear and pro-social emotions towards the cancer vignette compared to the depression vignette. Depression was associated with lower anger, but higher fear levels and higher levels of pro-social emotions compared to the healthy vignette, we found similar findings comparing the schizophrenia and the healthy vignettes. Focusing on understanding and willingness to support, we see similar results. Participants showed the most understanding and a higher willingness to support the person with cancer and the least for the healthy person. These findings are mostly consistent with our hypotheses and reflect current research findings.

A recent Australian study examined the differences for euthanasia for people who suffer from physical or mental illness [24]. They found more positive attitudes for individuals with cancer than for individuals with a mental health disorder (depression, schizophrenia). The effect was mediated by participants’ perceptions of autonomy and illness controllability. Even if euthanasia differs from PAS in its implementation, it is clear to see here that medical aid in dying is more likely to be accepted for physical than for mental illnesses. However, this is often based on the assumption that terminal illnesses are incurable and untreatable. In contrast, mental health disorders often have potential for successful treatment and symptom relief. Interestingly, participants in the study by Levin et al. [24] attributed a higher degree of autonomy to the cancer patient and less control over their own illness. This also reflects the assumption that individuals with mental health disorders may have impaired decision-making capacity due to their condition, and that the desire to die may be a symptom [8, 13]. Similar results were found in an Israeli study in attitudes towards euthanasia for patients with mental and physical illnesses [25]. The results showed a much more liberal attitude towards patients with cancer.

Looking at the attitudes of healthcare professionals, similar results can be seen. In a Canadian study assessing the attitudes from psychologists, the majority rejected PAS for mental health disorders, whereas they saw it as justified for terminal illness [17]. In another study from the Netherlands, over 80% of physicians stated they would grant a request for PAS or euthanasia for people with cancer and other physical illnesses [18]. In contrast, only 34% would do so for mental health disorders. Physical conditions might be more objectively measurable, while psychiatric conditions are not always visible and assessable from the outside making the acceptance of PAS in psychiatric cases more complex. Personal experiences with somatic or psychiatric illnesses might also influence attitudes towards PAS in these cases.

However, it should be noted that, due to various factors, the attitudes of doctors and laypeople are not fully comparable. The attitudes of doctors are sometimes influenced by their clinical training, a differentiated view of diseases and their experience in a clinical context. Understanding these discrepancies is essential for improving communication and aligning expectations between medical professionals and the general public.

Interestingly, we also found differences between the two mental health disorder vignettes. Participants showed more liberal attitudes towards the vignette with schizophrenia. In the study by Levin et al. [24] schizophrenia is perceived as less controllable than depression which might play a role in having a more accepting attitude for PAS. Another explanation could be that participants have experienced depression themselves and are thus aware of successful treatment options and have witnessed how the wish to die can change when depressive symptoms improve.

In our study, we also implemented a vignette displaying a healthy person. Participants showed significant lower levels of pro-social emotions towards this person as well as significant higher levels of anger. Participants were least able to understand the wish for PAS, stating that PAS should not be allowed and that the complaints were not severe enough. Healthy people seeking PAS are rather rare; in the Netherlands, for example, they account for 3% of requests [18]. There is no reliable data available for Germany on how many healthy individuals seek PAS. The reasons why healthy people want to make use of PAS are therefore unknown and cannot be conclusively clarified. In reports from right-to-die organizations, these cases are often categorized as ‘saturation with life’. Experts call for precise clarification and consideration of granting that request. However, there is also a significantly reduced willingness, at least among medical staff, to support healthy individuals who are tired of living in their request for PAS [18, 19].

However, the current findings should be considered in light of influencing factors. A person’s personal experiences inevitably shape their attitudes toward certain topics [26, 27]. While we have controlled for suicide loss, other experiences with illness may still influence attitudes toward PAS. Additionally, all participants were presented with the case vignettes in a specific order, which could have influenced their evaluation of each individual vignette. It is possible that the evaluations would have differed if the order had been changed, or if participants had only been exposed to one vignette at a time.

### Limitations

The results of this study provide important insights into attitudes towards PAS in the general population. However, the results should be considered against the background of some limitations. Firstly, our sample consists of over 90% women, which limits the generalizability. This gender imbalance may impact the generalizability of the results, especially in understanding how different demographics perceive PAS. Another limitation of this study is the potential self-selection bias inherent in voluntary participation. We cannot draw conclusions about causality based on our findings. While the study is cross-sectional, the main limitation for causal inference arises from the within-subjects design and the lack of random assignment, which restrict the ability to determine directional effects.

Although we used validated questionnaires, we also used some items that were not validated. The results must be interpreted considering this aspect. The evaluation of the differences in understanding and acceptance were analyzed at item level. It should be noted here that the analyses are therefore less meaningful and there is a higher probability of alpha error accumulation than by using sum scores and less tests. Another limitation is the development of the case vignettes. While these vignettes were designed to reflect the unique legal and cultural aspects of PAS in Germany, they were not based on pre-existing vignettes commonly used in similar studies. However, the use of standardized vignettes, which have been tested in other studies, might have allowed for easier comparisons with other populations and settings. In addition, the difference in sociodemographic information could possibly influence the attitudes on PAS in the respective case. One vignette involves a patient with active psychotic symptoms, which may impair decision-making capacity and would typically preclude PAS. It was included to explore responses to ethically complex scenarios, and findings should be interpreted accordingly, also considering the potential to enforce stigma. The vignette with the healthy person reports predominantly negative factors, which could have influenced the participants’ answers. While we provided information on the respective individual in each case vignette, specifics of their treatment, such as type, intensity and quality of care, were not included. Although the power analysis indicated a required sample size of 62, the final sample was substantially larger due to convenience sampling over a fixed period. While this may raise concerns about overpower, the larger sample increases the stability of estimates and allows for broader generalizability within the studied population.

Future research should aim for more balanced recruitment, particularly ensuring an equal gender ratio. Additionally, it is advisable to include participants’ experiences with various illnesses, as these could potentially influence their attitudes toward PAS. To minimize contrast effects, consideration should also be given to presenting the case vignettes in a randomized order.

## Conclusion

This study is among the first studies employing vignettes in examining emotional reactions and understanding of PAS across various illnesses as well as in healthy individuals in Germany. The findings are highly relevant to the ongoing discourse on PAS in Germany. Even though PAS has been permitted for four years now, there are still major uncertainties. The results provide valuable insights into the prevailing attitudes within the general population, highlighting potential concerns and perspectives. Additionally, the study identifies trends indicating the extent to individuals receive support for their request for PAS and the level of understanding they encounter. Nevertheless, the results underscore that PAS remains a significant social and ethical issue, which will continue to be discussed intensively in the future.

## Electronic supplementary material

Below is the link to the electronic supplementary material.

Supplementary Material 1

## Data Availability

The data can be requested from the first author.
