# Supplementary material for: Do mental or somatic diagnoses influence emotional response and perception of physician-assisted suicide in Germany? A vignette-based experiment
Source: BMC Med Ethics. 2025 May 15;26:62. doi: 10.1186/s12910-025-01223-3 (PMC12079990; doi:10.1186/s12910-025-01223-3)
Supplement: Supplementary file 1 — Supplementary Material 1 [file 12910_2025_1223_MOESM1_ESM.docx]

**Supplementary Material**

***Case Vignettes***

| Case Vignette 1: Mrs. Meier, Cancer |
| --- |
| Six months ago, 52-year-old Mrs. Meier was diagnosed with lung cancer after visiting her GP due to chest pains. She was immediately given chemotherapy, but the cancer was already too advanced and had already spread throughout her body. The doctors gave her a prognosis of around 9 months. Mrs. Meier is in constant severe pain, which can only be alleviated to a limited extent with pain medication. She is scared of the coming months, especially of needing care and of increasing shortness of breath. Her illness has already left her physically very limited and exhausted, so that she can hardly go about her everyday life and needs help. Mrs. Meier feels well looked after by the doctors treating her. Mrs. Meier would like to make use of assisted suicide in order to spare herself a longer period of suffering. She wishes to die in her own home as long as this is still possible. |

| Case Vignette 2: Mrs. Becker, Depression |
| --- |
| Mrs. Becker is 48 years old, married and has two children aged 20 and 25. She was first diagnosed with severe depression 8 years ago. Since then, she has felt depressed and drained almost all the time and finds it difficult to cope with everyday life. Sometimes she lies in bed for several days and barely manages to get up. She has already lost her job due to the illness and has since taken early retirement. Mrs. Becker has already undergone psychotherapy several times. She was also temporarily hospitalized in a clinic and in an inpatient rehabilitation clinic. After that, she only felt better for a short time. She has also tried several antidepressants, but none have brought about the desired improvement. In addition, she suffers from severe side effects such as headaches and dizziness due to the medication. For about 5 years, Mrs. Becker has repeatedly suffered from very urgent suicidal thoughts. In the last two years, she had already made two suicide attempts. She often experiences emotional pain and wishes that this suffering would stop. Mrs. Becker sees her situation as hopeless and has little hope that her condition will improve. In the meantime, Mrs. Becker is considering assisted suicide. |

| Case Vignette 3: Mr. Weber, no illness |
| --- |
| Mr. Weber is 67 years old and has been retired for six months. He lives alone after divorcing his wife 5 years ago. He has always had a full life, but increasingly has the feeling that he wants to end his life. He has always been very afraid of limitations due to illness in old age and the need for care. In addition, his mother has dementia and he is very afraid that he himself could develop it, as he has been able to observe the progression of the disease in his mother. He increasingly has the feeling that he has lived his life, which has been very satisfying for him up to now. He has given it a lot of thought and would like to make use of assisted suicide. It is important to him to die in a self-determined way and not to be dependent or in need of care in later life. |

| Case Vignette 4: Mr. Mueller, Schizophrenia |
| --- |
| Mr. Müller is 34 years old and was diagnosed with schizophrenia in his youth due to acoustic delusions. He was repeatedly hospitalized due to acute psychotic phases. During these phases, he heard various whispering voices commenting on his behavior, insulting him and threatening him. He experienced great anxiety at times and no longer dared to leave the house because of the voices. He also had the feeling that other people can read his mind, he could no longer think clearly in these situations and repeatedly reacted aggressively. He finds it increasingly difficult to distance himself from these voices and without high doses of medication, the condition is almost unbearable for him. Between these phases, he often feels empty, listless and emotionally numb. The therapeutic treatment options have now been exhausted, and although he continues to seek medical and psychotherapeutic support, his hallucinations will probably never completely subside, according to his therapists. Mr. Müller has long felt that his life is no longer worth living and wishes he could calm down and no longer have to endure this state. He has the impression that he is a great burden on his family. He is now thinking about taking his own life by assisted suicide. |

**Items**

***Understanding***

|  | Completely disagree |  |  |  | Completely agree |
| --- | --- | --- | --- | --- | --- |
| 1. I can understand Mrs. Meier's wish for assisted suicide. | 🞏 | 🞏 | 🞏 | 🞏 | 🞏 |
| 1. I think Mrs. Meier should make use of further therapy options. | 🞏 | 🞏 | 🞏 | 🞏 | 🞏 |
| 1. I do not find Mrs. Meier's illness/complaints serious enough to make use of assisted suicide. | 🞏 | 🞏 | 🞏 | 🞏 | 🞏 |
| 1. Mrs. Meier should not be allowed to die through assisted suicide. | 🞏 | 🞏 | 🞏 | 🞏 | 🞏 |
| 1. I think assisted suicide should generally be allowed for people with this diagnosis. | 🞏 | 🞏 | 🞏 | 🞏 | 🞏 |
| 1. If I were in Mrs. Meier's situation, I would also think about assisted suicide. | 🞏 | 🞏 | 🞏 | 🞏 | 🞏 |

***Willingness to Support***

*If Mrs. Meier were a person close to you, then...*

|  | Completely disagree |  |  |  | Completely agree |
| --- | --- | --- | --- | --- | --- |
| 1. …I would support her in her plans. | 🞏 | 🞏 | 🞏 | 🞏 | 🞏 |
| 1. ….I would propose alternatives. | 🞏 | 🞏 | 🞏 | 🞏 | 🞏 |
| 1. … I would be furious about her decision. | 🞏 | 🞏 | 🞏 | 🞏 | 🞏 |
| 1. ...I would advise her not to do this. | 🞏 | 🞏 | 🞏 | 🞏 | 🞏 |
| 1. …I would accompany her to counseling sessions. | 🞏 | 🞏 | 🞏 | 🞏 | 🞏 |
| 1. … I would distance myself from her because of her wish. | 🞏 | 🞏 | 🞏 | 🞏 | 🞏 |
